# Simplified Multiple-Well Approach for the Master Equation Modeling of Blackbody Infrared Radiative Dissociation of Hydrated Carbonate Radical Anions

**DOI:** 10.1021/jacs.2c07060

**Published:** 2022-11-16

**Authors:** Magdalena Salzburger, Milan Ončák, Christian van der Linde, Martin K. Beyer

**Affiliations:** Institut für Ionenphysik und Angewandte Physik, Universität Innsbruck, Technikerstraße 25, 6020Innsbruck, Austria

## Abstract

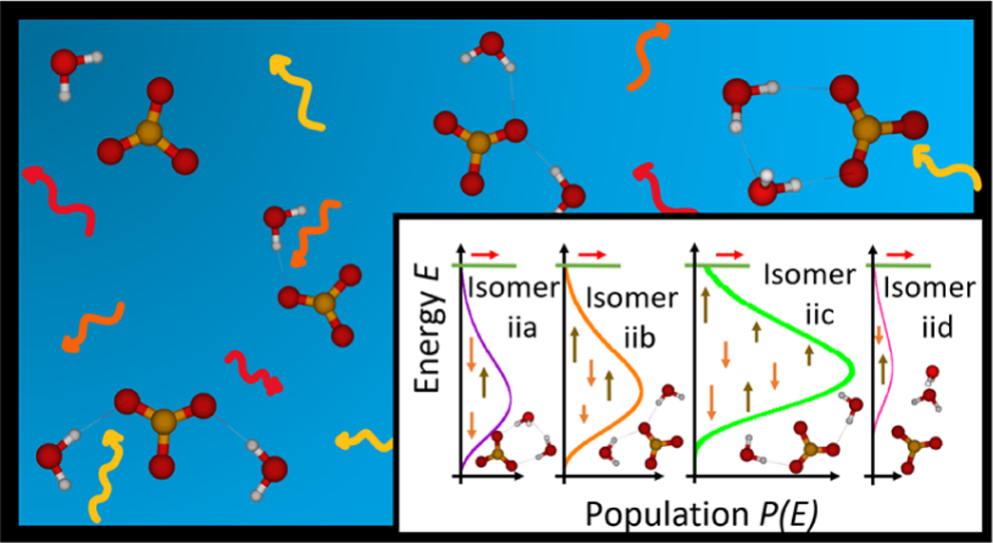

Blackbody infrared radiative dissociation (BIRD) in a collision-free environment is a powerful method for the experimental determination of bond dissociation energies. In this work, we investigate temperature-dependent BIRD of CO_3_^·–^(H_2_O)_1,2_ at 250–330 K to determine water binding energies and assess the influence of multiple isomers on the dissociation kinetics. The ions are trapped in a Fourier-transform ion cyclotron resonance mass spectrometer, mass selected, and their BIRD kinetics are recorded at varying temperatures. Experimental BIRD rates as a function of temperature are fitted with rates obtained from master equation modeling (MEM), using the water binding energy as a fit parameter. MEM accounts for the absorption and emission of photons from black-body radiation, described with harmonic frequencies and infrared intensities from quantum chemical calculations. The dissociation rates as a function of internal energy are calculated by Rice–Ramsperger–Kassel–Marcus theory. Both single-well and multiple-well MEM approaches are used. Dissociation energies derived in this way from the experimental data are 56 ± 6 and 45 ± 3 kJ/mol for the first and second water molecules, respectively. They agree within error limits with the ones predicted by ab initio calculations done at the CCSD(T)/aug-cc-pVQZ//CCSD/aug-cc-pVDZ level of theory. We show that the multiple-well MEM approach described here yields superior results in systems with several low-lying minima, which is the typical situation for hydrated ions.

## Introduction

Molecular clusters CO_3_^·–^(H_2_O)_1,2_ are found in the lower region of the ionosphere^1^ and in the troposphere.^2^ These clusters also occur in the ionosphere of Mars.^3^ Direct sampling of the air confirmed the presence of CO_3_^·–^ in the boreal forest.^4^ In the condensed phase, CO_3_^·–^ plays a role as a radical in biological processes.^5^ Of particular interest is the potential role of CO_3_^·-^(H_2_O)_1,2_ in catalytic cycles in atmospheric chemistry. For example, CO_3_^·–^ is involved in the electron-mediated oxidation of formic acid with ozone.^6^

Since CO_3_^·–^ has been detected in the D-region of the ionosphere in 1971,^7,8^ properties of the molecule have been investigated in detail. Quantum chemical calculations yield an electron affinity of 3.85–4.08 eV for CO_3_^·–^.^9^ Calculations at the B3LYP/6-311++G(d,p) level of theory were done to assess the structure, energies, and zero point energies of the most stable structures of CO_3_^·–^(H_2_O)_1–8_.^10^ The structure of singly and doubly hydrated CO_3_^·–^ was studied with infrared multiple photon dissociation spectroscopy by our group.^11^ A comparison of the calculations with the experimental spectra shows that several isomers of CO_3_^·–^(H_2_O)_1,2_, which are close in energy, contribute to the spectrum. At a temperature of 85 K, these isomers interconvert, indicating considerable mobility of the hydrating water molecules.^11^

Binding energies of H_2_O to CO_3_^·–^ were determined by Castleman et al.^12,13^ using high-pressure mass spectrometry (HPMS). Armentrout et al. refined collision-induced dissociation as a useful method for the determination of binding energies.^14−16^ Blackbody infrared radiative dissociation (BIRD) is a well-established technique to investigate unimolecular dissociation in collision-free environments.^17−28^ Studied species include hydrated atomic and molecular ions,^29−35^ molecular complexes,^26,36,37^ and biomolecular ions.^38−40^ For BIRD measurements, ions are trapped under ultra-high vacuum (UHV) conditions, usually for several seconds. During this trapping time, photons from ambient blackbody infrared radiation can be absorbed, and the ions in turn emit infrared photons. If the energy content of the ions rises above the energetic threshold, dissociation is possible. Dunbar described the key concepts of BIRD in a seminal review.^25^

The size of the molecular or cluster ion and the dissociation energy determine the most suitable model for the description of the BIRD process. If the energy exchange via emission and absorption of infrared photons is much faster than dissociation, termed the rapid energy exchange (REX) limit by Williams,^41^ a simple Arrhenius plot can be used to yield activation energies.^18^ The REX limit is reached by systems with many internal degrees of freedom and relatively high binding energies, which need significant excess energy above the threshold for dissociation. Very small molecules, which decay immediately as soon as the threshold energy is reached, can be modeled using a truncated Boltzmann distribution. For systems of intermediate size, master equation modeling (MEM) must be used to obtain meaningful quantitative results,^22^ but it also works for other cases.

MEM is described, for example, by Williams et al.^22,40,42^ It relies on a set of differential equations describing the time evolution of the system.^22^ The initial internal energy distribution of the population is divided into narrow energy bins. The time evolution of the population is described, considering that ions can switch their bins due to emission or absorption of photons. Dissociation rate constants at a given energy bin are calculated by statistical methods, like Rice–Ramsperger–Kassel–Marcus (RRKM) theory or variational transition state theory.^36^

Usually, only the isomer with the lowest energy, the global minimum structure, is used for MEM.^26,29^ In this work, we use a combination of BIRD experiments and a new multiple-well approach for MEM to get experimental binding energies for the first and second water molecules to CO_3_^·–^. Comparison with high-level ab initio calculations indicates that the used approach is well suited for the description of BIRD of hydrated carbonate ions.

## Methods

### Experimental Section

BIRD kinetics were measured using a 4.7 Tesla Fourier-transform ion cyclotron resonance mass spectrometer (FT-ICR MS) in the temperature range of 250–330 K. The investigated clusters were generated with a gas mixture of O_2_, CO_2_, H_2_O, and the carrier gas He in the hot plasma of a laser vaporization source, followed by supersonic expansion.^11,43^ The molecular clusters were in the electronic ground state due to the supersonic expansion; their vibrational temperature is below room temperature upon leaving the ion source.^44^ The ions were then guided to the ICR cell, where they were mass selected and stored in UHV conditions. The temperature of the ICR cell walls can be regulated with a variable supply of liquid nitrogen^45^ or warm water. Only a small part of the solid angle (0.2%) is not covered by the temperature regulation system; this part allows for introducing black-body radiation at a temperature of ∼288 K and the temperature of the cooling water around the vacuum tube in the magnet.

A collision-free environment in the ICR cell is crucial for BIRD measurements.^25^ The pressure in the ICR cell was in the range of 10^–10^ mbar at low temperatures but reached the 10^–9^ mbar regime when the ICR cell was brought to higher temperatures. Only measurements with a pressure in the low 10^–10^ mbar region were used for data analysis because measurements at higher pressure resulted in systematically too high reaction rates, indicating collisional activation. Also, the measured rates exhibit some day-to-day variation. This may be due to a small number of atoms and molecules leaking through the piezo valve, which result in a neutral molecular beam that overlaps with the ion cloud and leads to collisions. Therefore, only the kinetics of the measurement day with the lowest resulting rates were considered. Both problems primarily affected CO_3_^·–^(H_2_O) due to its extremely long BIRD lifetime. A comparison of all data with the selected data is given in the Supporting Information (Figure S1, Table S1).

### Quantum Chemical Calculations

Quantum chemical calculations were performed using Gaussian.^46^ Ground-state isomeric structures and vibrational modes of CO_3_^·^^–^(H_2_O)_1,2_ clusters were calculated at the CCSD/aug-cc-pVDZ level of theory and have already been reported elsewhere.^11^ Additionally, single-point calculations of the electronic energies at the CCSD(T)/aug-cc-pV*X*Z, *X* = D, T, and Q level of theory were performed, employing the zero-point correction of the CCSD/aug-cc-pVDZ calculation. Relative energies obtained at the CCSD(T)/aug-cc-pVQZ level were used for all further calculations in this paper. Results using extrapolation to the complete basis set limit (CBS) using TZ and QZ basis sets are collected in Table S2. All calculations were done at 0 K. For zero-point correction and RRKM calculations, vibrational frequencies were scaled with 0.96, as recommended by NIST.^47^

### MEM

The goal of MEM is to model the dissociation rate for given temperatures and to derive activation energies from a fit of the modeled dissociation rates to experimental data. At the available energies in the near-ambient temperature black-body radiation field, all cluster ions are in the electronic ground state, while ro-vibrational levels are statistically populated. For the description of the thermal population as well as for the RRKM calculations, we use rigid rotor harmonic oscillator approximation. The clusters are approximately symmetric tops. Angular momentum conservation is accounted for by treating the 2D-rotor as inactive.^15^ The rotational density of states is calculated from the 1D-rotor that corresponds to quantum number *K*, without limitations in the value of *K*, using the formalism described by Gilbert and Smith.^48^ It has been discussed previously that this introduces negligible error for systems with small rotational constants.^15^ All internal degrees of freedom are treated as harmonic oscillators.

In the previously used single-well approach,^25^ it is assumed that all molecules are in the global minimum on the potential energy surface. In the simplified multiple-well approach described here, additional low-lying local minima are allowed to be populated, aiming at a better representation of the accessible phase space of the system. Since the barriers for interconversion of the isomers are well below the dissociation threshold, equilibrium between the isomers is reached rapidly at the timescale of ps. The relative population of isomers at a given energy can thus be calculated from their densities of state.

**Figure 1:**
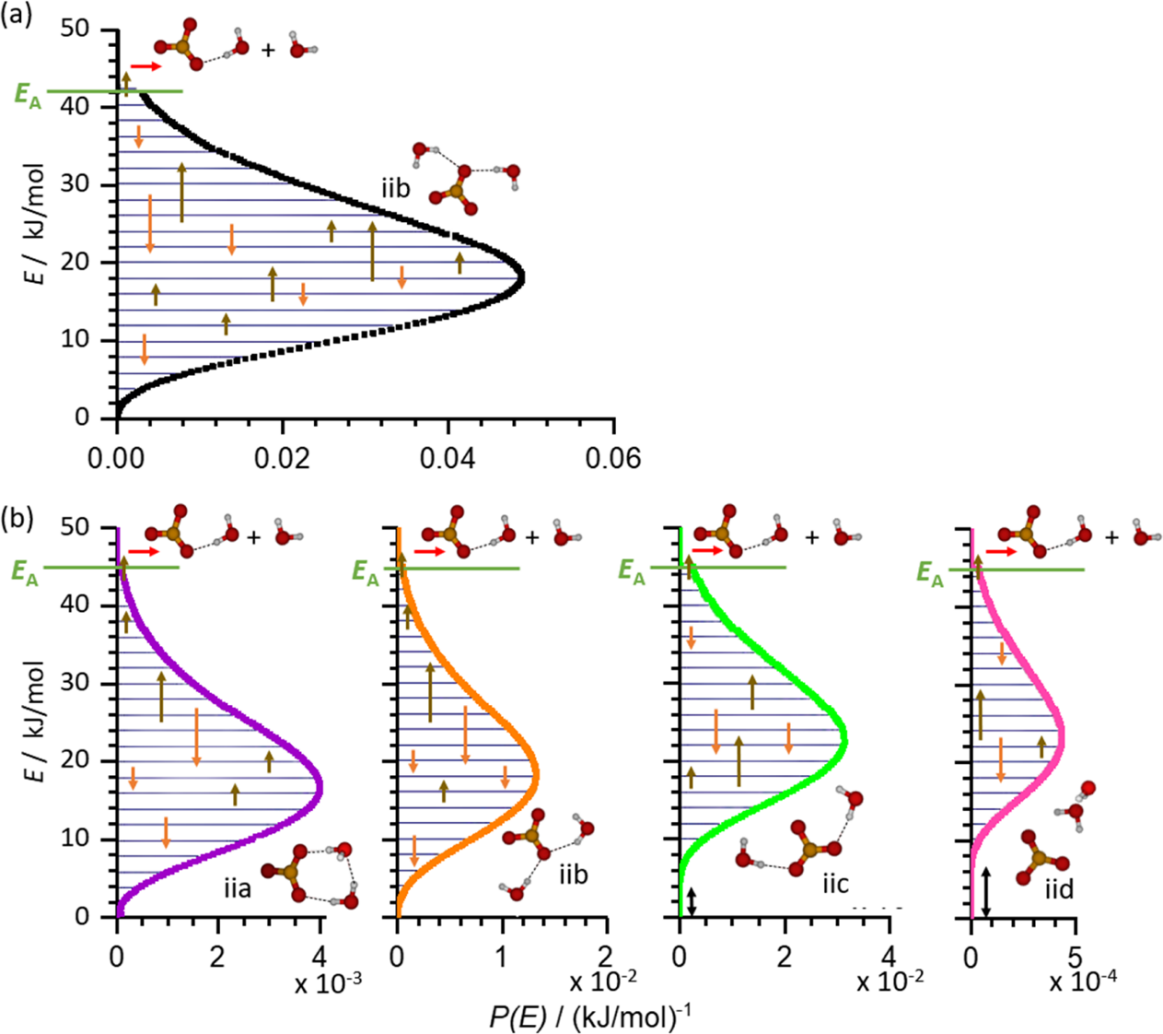
Illustration of the (a) single-well approach and (b) multiple-well approach in MEM. The population *P*(*E*) at 310 K is shown on the *x*-axis as a function of the energy *E* on the *y*-axis. The horizontal lines indicate energy bins, which are shown to be 10 times larger than in the actual simulation. The activation energy *E*_A_ is marked with a green line. Brown, orange, and red arrows illustrate photon absorption, emission, and dissociation, respectively. The bold black arrows show the relative shift in the energy of an isomer compared to other isomers.

The MEM protocol contains the following steps:

1 In the beginning, a Boltzmann distribution is assumed for the population of different isomers *i* within discrete energy bins *b*. In the multiple-well approach, the population is distributed over all isomers, and each isomer has its own Boltzmann distribution (see Figure 1). For the modeling, the distribution is divided into narrow energy bins of width Δ*E* = 20 cm^–1^. The initial population *P*_*i*,*b*_ of a given isomer *i* and energy bin *b* are obtained via eq 1.

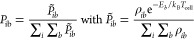
1Here, *E*_*b*_ = *b*Δ*E* is the energy of bin *b* relative to the vibrational ground state of the global minimum, *k*_B_ is Boltzmann’s constant, and *T*_cell_ is the temperature of the ICR cell.  is the population before normalization, and *P*_*i*,*b*_ is normalized. The density of states ρ_*ib*_ for the given isomer and bin is calculated with the Beyer–Swinehart algorithm^49^ in the version described by Gilbert and Smith for the ro-vibrational density of states.^48^ Information about ground-state electronic energies, harmonic vibrational frequencies, and rotational constants is taken from quantum chemical calculations. Finally, the population is normalized.

2 Population is transferred from bin *b* to bin *c* via photon absorption for *b* < *c* and photon emission for *b* > *c*, as illustrated in Figure 1. The radiative loss rates *k*_abs,*ibc*_, *k*_em,*ibc*_ are calculated for bin *b* of isomer *i* by summing over the vibrational modes *q* that fulfill the condition , eqs 2 and 3.

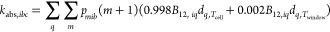
2
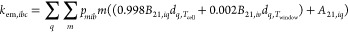
3Here, *A*_21,*iq*_ is the Einstein coefficient of mode *q* for spontaneous emission, and *B*_12,*iq*_ and *B*_21,*iq*_ are the corresponding coefficients for stimulated absorption and emission, respectively.  is the radiation density^18^ at ICR cell temperature, and  is the radiation density at the temperature of the ICR cell window. The window covers 0.2% of the solid angle. The probability *p*_*mib*_ that the vibrational mode *q* of isomer *i* is *m* times populated in bin *b* is calculated with the density of states ρ_*ib*_ and the contribution of mode *q* populated *m* times to the density of states ρ_*mib*_.
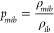
4

The Einstein coefficients for spontaneous emission and for induced processes multiplied with the radiation densities are given by
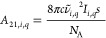
5
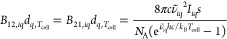
6
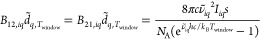
7where *c* is the speed of light,  is the wavenumber of the vibrational mode *q* in m^–1^ of isomer *i*, *I*_*iq*_ is the IR intensity in m/mol, *N*_A_ is Avogadro’s constant, *h* is Planck’s constant, *k*_B_ is Boltzmann’s constant, *T*_cell_ is the temperature of the ICR cell, *T*_window_ is the temperature of the ICR cell window, and *s* is an empirical intensity scaling factor for IR intensities. This scaling factor is an adjustable parameter, which mostly accounts for the error introduced by the harmonic approximation. The factor is not frequency dependent and will be discussed in more detail below.

A third process, dissociation, occurs if the internal energy of the system exceeds the activation energy *E*_*i*_^⧧^, which, in this case, is equal to the water binding energy. The dissociation is assumed to occur along a proper vibrational mode. The dissociation rate *k*_diss,*ib*_ for isomer *i* and bin *b* is calculated using RRKM theory
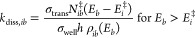
8

Here, *h* is Planck’s constant and *N*_*ib*_^⧧^ is the sum of states within bin *b* at the transition state between isomer *i* and the products. Again, ρ_*ib*_ and *N*_*ib*_^⧧^ are calculated with the Beyer–Swinehart algorithm^49^ as described by Gilbert and Smith for the ro-vibrational sum of states.^48^ In the present case, the products after dissociation are identical for all isomers, and thus, *E*_*i*_^⧧^ also does not depend on the isomer. σ_well_ and σ_trans_ are degeneracies of the well and transition states, respectively, i.e., the products of spin degeneracy, path degeneracy, and degeneracy for optical isomers. Rotational symmetry is accounted for in the calculation of the ro-vibrational sum and densities of state, as described by Gilbert and Smith.^48^

3 The iterative change in population is numerically evaluated with finite time steps Δ*t.* However, emission, absorption, and dissociation rates span several orders of magnitude. Therefore, a standard finite-element approach treating Δ*t* as a differential time step is not possible. Instead, the integrated first-order rate law with exponential decay functions needs to be employed. This makes evaluation a bit more complicated but quite precise. In the first step, the loss of population Δ*P*_*ib*_^–^ of isomer *i* in bin *b* is calculated.

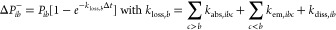
9

The gain Δ*P*_*ib*_^+^ is then obtained via the population transferred from other bins by photon emission or absorption.
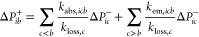
10

The net population change Δ*P*_*ib*_ of isomer *i* in bin *b* amounts to
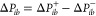
11

Dissociation leads to a net loss of population Δ*P*, which yields the BIRD rate *k*_BIRD_.
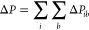
12
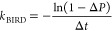
13

To obtain the new population, we first calculate the total population *P*_tot,*b*_(*t* + Δ*t*) of bins *b* by applying the changes to the population and normalization. *P*_tot,*b*_(*t* + Δ*t*) is then distributed over the isomers according to their densities of state to yield *P*_*ib*_(*t* + Δ*t*).
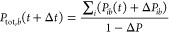
14
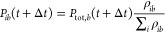
15

4 After some simulation time, a stationary state is reached in which the population does not change significantly during Δ*t*, and the BIRD rate *k*_BIRD_ converges to a constant value. This way, dissociation rates *k*_BIRD_(*T*) are calculated with a fixed *E*^⧧^ for different temperatures. The calculated rates are then plotted in the Arrhenius plot and compared with the experimental results.

The whole procedure is repeated for different *E*^⧧^. A change in *E*^⧧^ leads to a change in the slope of the resulting Arrhenius plot. Therefore, *E*^⧧^ can be used as a fit parameter by fitting the slope of the modeled Arrhenius plot to the slope of the experimental Arrhenius plot to obtain *E*^⧧^ of the given process. Deviations between the calculated and real infrared intensities *I*_*iv*_ lead to an offset of the modeled plot from the experimental data, which is corrected with an empirical scaling factor *s*, a second fit parameter.

## Results and Discussion

### Experimental Results

For both clusters, CO_3_^·–^(H_2_O) and CO_3_^·–^(H_2_O)_2_, heating by ambient blackbody infrared radiation results in the evaporation of water molecules. For CO_3_^·–^(H_2_O)_2_, we observe a sequential loss of both water molecules. For the investigation of weakly bound clusters, as in our case CO_3_^·–^(H_2_O)_2_, which dissociate rapidly at room temperature, the ICR cell has to be cooled to make dissociation rates suitable for quantitative BIRD experiments.^45^ On the other hand, for CO_3_^·–^(H_2_O), temperatures higher than room temperature were needed.

**Figure 2:**
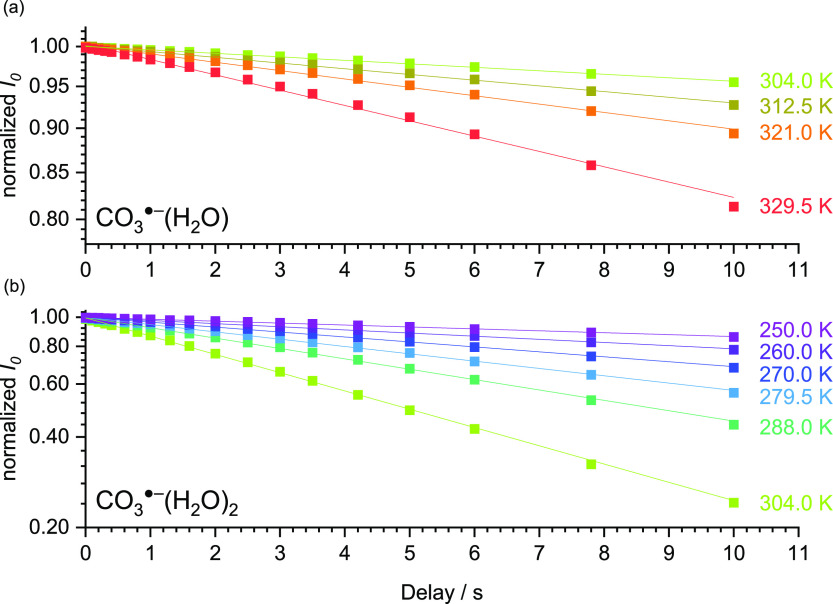
Unimolecular BIRD kinetics of (a) CO_3_^·–^(H_2_O) and (b) CO_3_^·–^(H_2_O)_2_. The intensity of the reactant normalized to the total ion intensity *I*_0_ is plotted as a function of the delay. Note the semilogarithmic scale in both panels. The data are fitted with exponentials shown with lines.

Figure 2 shows the results of the measured BIRD kinetics of mass-selected CO_3_^·–^(H_2_O) and CO_3_^·–^(H_2_O)_2_, with the reactant intensity normalized to the total intensity shown as a function of delay. For the total intensity, the parent and product ion intensities are summed, considering in the case of CO_3_^·–^(H_2_O)_2_ also the consecutive production of CO_3_^·–^. As expected, evaporation of a water molecule is faster for higher temperatures and faster for the doubly hydrated carbonate ion. The linear graphs in the semilogarithmic plots show that BIRD kinetics is first order. There is no evidence of an induction delay, indicating that the ions rapidly reach a stationary state of absorption and emission of black-body radiation and water evaporation in the thermal radiation field of the ICR cell. The kinetics of CO_3_^·–^(H_2_O) at a temperature of 329.5 K exhibit a slightly systematic curvature, indicating that collisions may already be relevant. At higher temperatures, it becomes more and more challenging to provide a collision-free environment because water desorbs from the warm surfaces of the ICR cell. Due to the evident contribution of collisions, kinetics measured at temperatures above 329.5 K could not be used for quantitative evaluation.

**Figure 3:**
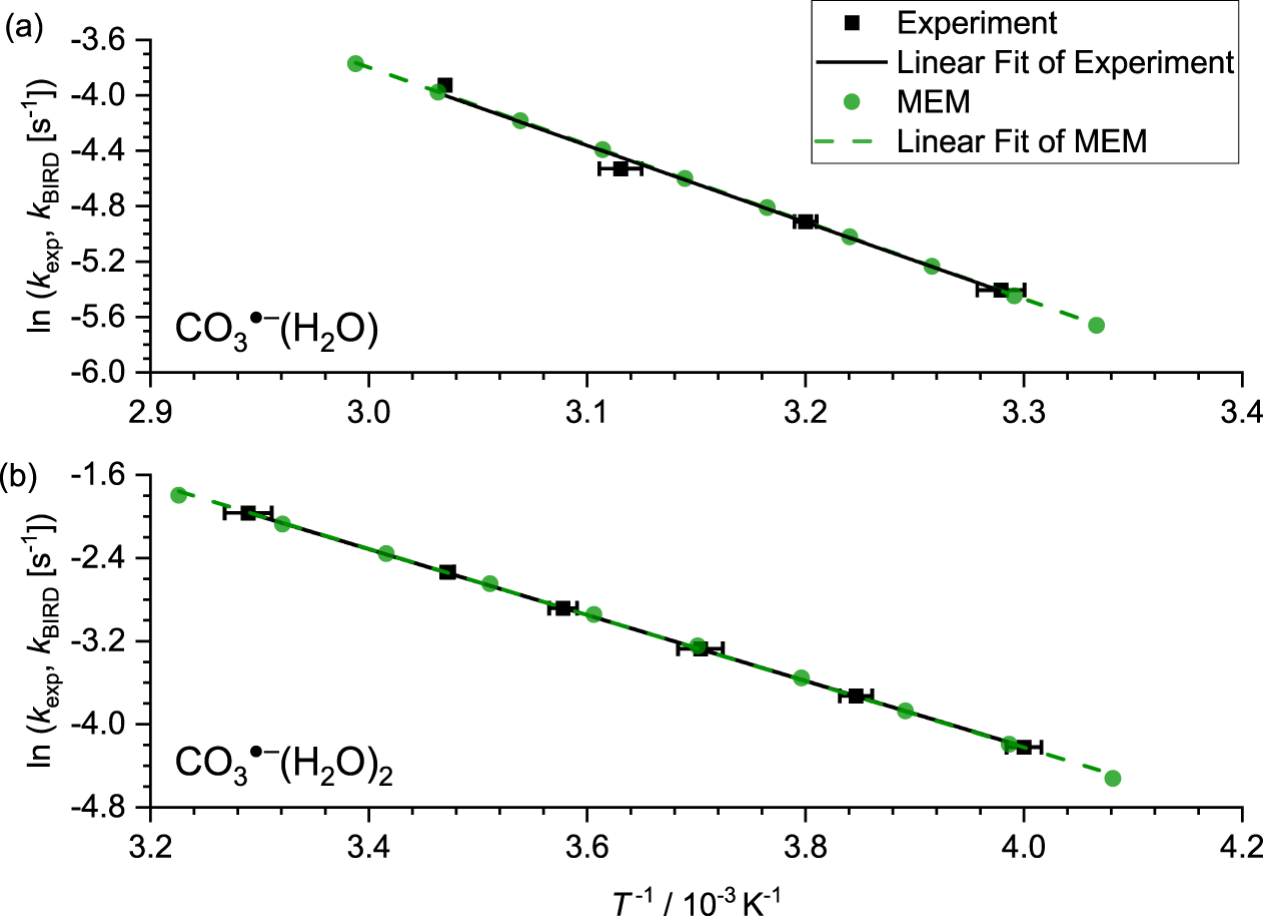
Arrhenius plot with experimental results shown in black; results of the MEM simulations in green. Error bars of experimental results account for temperature fluctuations during the experiment and the statistical error of the exponential fit to the kinetics. Note that these uncertainties of the rate constants are about 1–2% and thus hidden by the symbols.

The kinetics were fitted with an exponential function to obtain unimolecular reaction rate constants *k*_exp_. The statistical error of this fit is in the range of 1–2% and is used as an uncertainty for the rate constant. Systematic errors are largely due to the error in the temperature measurement, which is considered separately. This statistical uncertainty is much smaller than the typical 20–30% uncertainty of bimolecular rate coefficients because BIRD rate constants do not rely on the measurement of absolute pressure. The resulting rate constants are used for the Arrhenius plots in Figure 3. A linear fit yields apparent Arrhenius activation energies of 46 ± 3 kJ/mol for CO_3_^·–^(H_2_O) and 26.4 ± 0.1 kJ/mol for CO_3_^·–^(H_2_O)_2_. The corresponding Arrhenius pre-exponential factors are on the order of 10^5^ and 10^3^, respectively. These are far from the expected value for direct cleavage, which means that the studied systems are not in the REX limit.^42^ Since the REX limit is not reached, these apparent Arrhenius activation energies do not correspond to the water binding energies.

## Results of MEM

**Figure 4:**
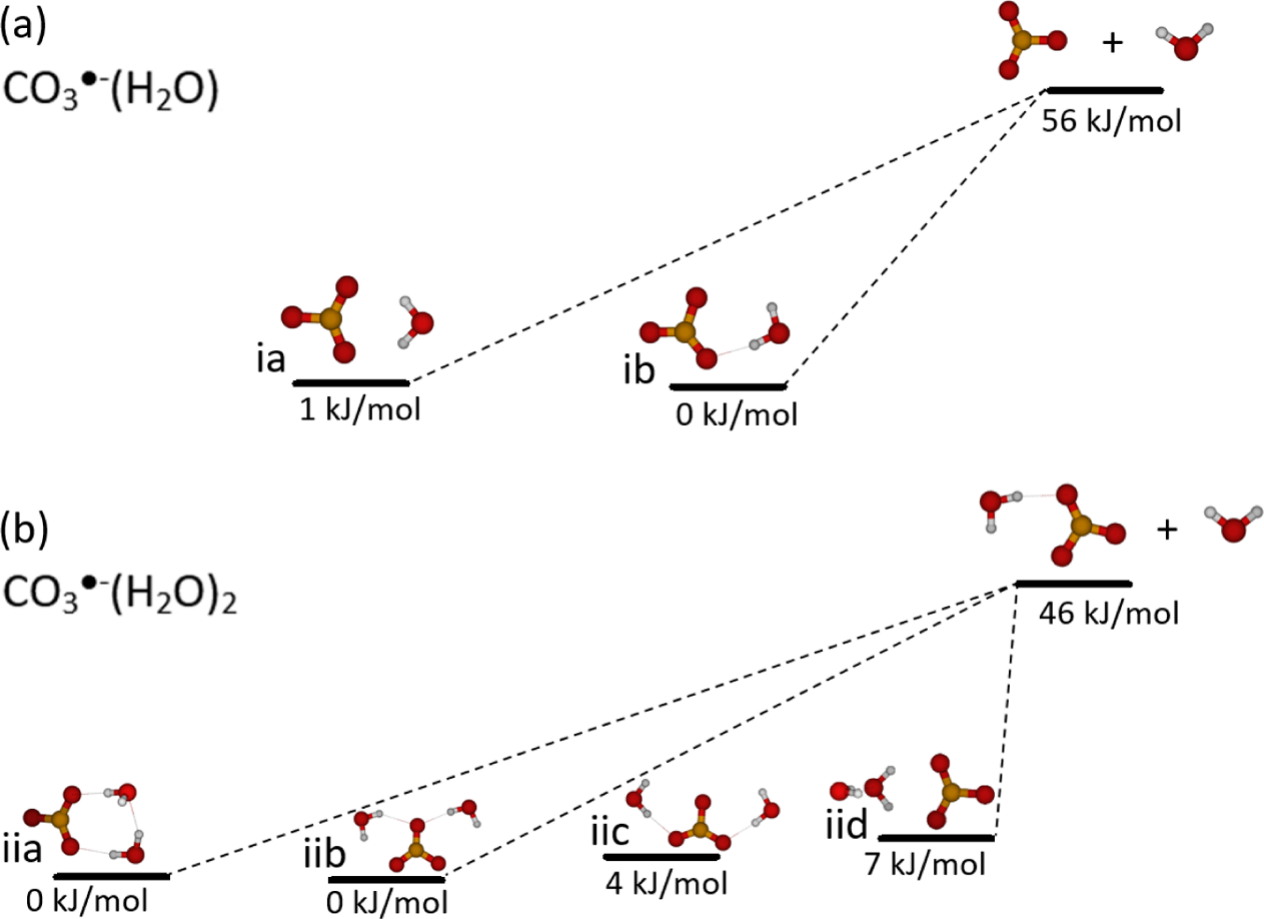
Allowed dissociation pathways for MEM simulations in (a) CO_3_^·^^–^(H_2_O) and (b) CO_3_^·^^–^(H_2_O)_2_. Calculated at the CCSD(T)/aug-cc-pVQZ//CCSD/aug-cc-pVDZ level at 0 K including zero-point energies, see the Methods section.

The isomers considered in the multiple-well approach and their relative energies, together with the dissociation asymptote, are illustrated in Figure 4. Direct dissociation of the water molecules without switching first between the different isomers is assumed to be possible. The barrier for interconversion between the isomers was previously calculated to lie at 6–10 kJ/mol,^11^ considerably below the dissociation energy; thus, isomerization is fast compared to dissociation, and the relative population of the isomers can be calculated from the density of states, eq 15. Rate constants modeled with the multiple-well approach for MEM are shown in green in Figure 3.

The activation energy and the empirical scaling factor were adapted to reach the same gradient and offset as the Arrhenius plot of the experiment, which yields activation energies and the corresponding empirical scaling factors for both CO_3_^·^^–^(H_2_O) and CO_3_^·^^–^(H_2_O)_2_.

Table 1 shows the resulting activation energies and empirical scaling factors. MEM activation energies are consistent with the dissociation energy resulting from ab initio calculations. The single-well approach and the multiple-well approach reveal the same results for CO_3_^·^^–^(H_2_O), where both considered isomers are almost isoenergetic, Figure 4. However, in the case of CO_3_^·^^–^(H_2_O)_2_, where the isomers differ in energy, the multiple-well approach results in a higher activation energy, closer to the calculated value.

**Table 1:** MEM Activation Energies *E*^⧧^ and Dissociation Energies *E*_dis_ at *T* = 0 K Calculated at the CCSD(T)/aug-cc-pVQZ//CCSD/aug-cc-pVDZ Level (in kJ/mol) and Literature Enthalpies Δ*H*_HPMS_^o^ from High-Pressure Mass Spectrometry^13^^,^^a^

	*E*^⧧^			
	single-well approach	multiple-well approach	*E*_dis_	Δ*H*_HPMS_^o^
CO_3_^·-^(H_2_O)	56 ± 6(*s* = 5.7)	56 ± 6(*s* = 5.7)	56	59 ± 1
CO_3_^·-^(H_2_O)_2_	42 ± 3(*s* = 1.2)	45 ± 3(s = 1.3)	46	57 ± 2

a The empirical scaling factor s is given in parentheses. Usage of CCSD(T)/CBS energies leads to negligible changes (Table S3). For the single well approach, only the most stable isomeric structure, **ib** and **iib**, is used.

The empirical scaling factor *s* is used in our simulation to adjust the offset of simulated dissociation rate constants. For CO_3_^·^^–^(H_2_O), this correction factor is higher than for CO_3_^·^^–^(H_2_O)_2_, where it is only slightly above 1. There are two reasons for an empirical scaling factor: (i) it accounts for all simplifications in the MEM like the harmonic approximation for frequencies and IR intensities of vibrationally excited modes, additional isomeric structures which might occur in the experimental population, the treatment of hindered rotations as vibrations, and the neglect of overtones and combination bands; (ii) a small change in the slope of the experimental Arrhenius plot has a small influence on the resulting activation energy but an exponential influence on the scaling factor, see Figure S2. A high scaling factor therefore may indicate that the experimental slope is slightly too high.

A possible reason for this deviation may be collisions, which occur more frequently at higher temperatures and thus lead to a systematically overestimated *k*_exp_. These arguments apply to CO_3_^·^^–^(H_2_O), which features experimental BIRD rate constants *k*_exp_ on the order of 0.01 s^–1^, similar to the collision frequency at 10^–10^ mbar. For CO_3_^·^^–^(H_2_O)_2_, the experimental Arrhenius plot exhibits next to no scattering, and the BIRD rate constants are more than an order of magnitude higher at a given temperature. Moreover, the temperature regime used for the BIRD kinetics of CO_3_^·^^–^(H_2_O)_2_ goes from room temperature down to 250 K, which leads to a cryopumping effect that further reduces collisions. All things considered, the experimental data base for CO_3_^·^^–^(H_2_O)_2_ is even more robust than that for CO_3_^·^^–^(H_2_O).

At this time, no definitive conclusion on the origin of the high scaling factor can be drawn. In any case, the thermochemically relevant information lies in the slope of the Arrhenius plot and not in the offset of the data. The scaling factor modifies the rate of photon absorption and emission in the same way, which results in a corresponding change in the simulated BIRD rate *k*_BIRD_. In the Arrhenius plot, this in turn translates into a constant offset, while the slope of the Arrhenius plot is determined exclusively by the activation energy *E*^⧧^. In Figure S2, we systematically change *E*^⧧^ in steps of 3 kJ mol^–1^ and adjust the scaling factor for best agreement with the experiment. The total uncertainty of the found binding energies is estimated to be ±6 kJ/mol for CO_3_^·^^–^(H_2_O) and ±3 kJ/mol for CO_3_^·^^–^(H_2_O)_2_.

**Figure 5:**
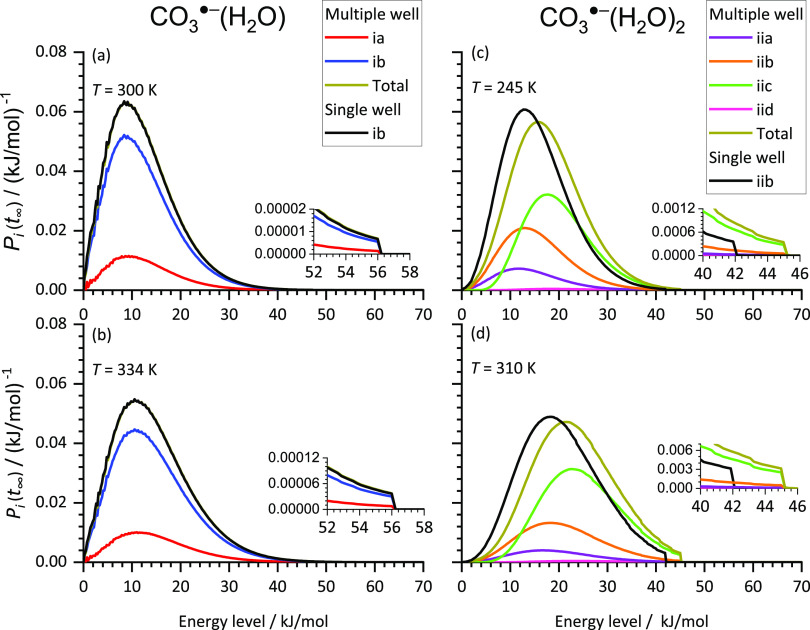
Population *P*_*i*_(*t*_∞_) of isomers and energy levels in the MEM stationary state in single-well and multiple-well approaches for comparison. Populations of CO_3_^·^^–^(H_2_O) are shown at a temperature of (a) 300 and (b) 334 K of CO_3_^·^^–^(H_2_O)_2_ at (c) 245 and (d) 310 K. For CO_3_^·^^–^(H_2_O), the sum of both isomers of the multiple well approach is not visible as it overlaps with the points of the single well approach.

An analysis of populated energy levels and isomers in the MEM stationary state provides additional insights. For the simulations of the populations shown in Figure 5, the activation energies and empirical scaling factors from Table 1 are used. As can be seen, the populations overall exhibit the behavior of a truncated Boltzmann distribution. Due to the small size of the studied systems, dissociation takes place right at the energetic threshold. Already in the first energy bin above the dissociation threshold, the RRKM lifetimes of the systems are below 1 ms, which is small compared to the timescale of the ICR experiment and the BIRD lifetimes. Kinetic shift effects are therefore negligible. A higher temperature leads to a shift in the population to higher energy levels. Together with the increased flux of black body radiation, this leads to the observed increase in the dissociation rate *k*_BIRD_ with temperature.

Populations simulated with the multiple-well approach are separated into the contribution of the individual isomers. Additionally, the sum of all contributions is shown in Figure 5. For CO_3_^·^^–^(H_2_O), both isomers are populated considerably. The energy distribution in the population is similar for both isomers and does not differ much from the single-well approach. The sum of both isomers in the multiple-well approach is almost the same as the population given by the single-well approach. Therefore, considering only the lowest-lying isomer for MEM gives reasonable results in this case. The population of CO_3_^·^^–^(H_2_O)_2_ is shown in panels (c) and (d). Here, the sum of all isomers shifts to higher energy levels compared to the single well approach, as the energetically higher-lying isomer **iic** dominates the population at higher energy levels. This is due to the low-lying vibrational modes of isomer **iic**, which result in a higher density of states than the other isomers. Isomer **iid**, in contrast, is too high in energy and therefore almost not populated. As a consequence, dissociation occurs predominantly from **iic**, and the fit of the simulated dissociation rates to the experimental dissociation rates results in a higher activation energy than with the single-well approach.

The single well approach could also be performed using a higher lying minimum instead of the global minimum. If the energy of the used isomer is near the global minimum, as is the case for **ia** or **iia**, this would not induce a considerable difference. In contrast, a single well approach using only isomer **iic** would give results near to the multiple-well approach (as **iic** is the most populated isomer according to the multiple-well approach), while using isomer **iid** would result in very high activation energies.

The high population of isomer **iic** for doubly hydrated carbonates can be explained qualitatively by the water molecules forming only one hydrogen bond, while in isomers **iia** and **iib**, at least one water molecule forms two hydrogen bonds. The out-of-plane vibrational mode of **iic** lies at only 2.7 cm^–1^ due to its vanishingly small force constant. The overall softer character of **iic** also lowers other vibrations, which in sum results in a significantly higher density of states of **iic** at a higher internal energy than other isomers. This remains true even if we artificially shift the 2.7 cm^–1^ vibration to 20 cm^–1^. Interestingly, isomer **iid**, with one water molecule in the second solvation shell, does not feature such an extreme low-frequency mode. Due to its higher relative energy, **iid** is not significantly populated.

Water binding energies to CO_3_^·^^–^ obtained by HPMS agree within error limits for CO_3_^·^^–^(H_2_O) with our calculations and MEM results, but for CO_3_^·^^–^(H_2_O)_2_, they are more than 10 kJ mol^–1^ higher (Table 1). This deviation clearly lies outside the error limits of both the experiment and theory. We tried several theory levels and did extensive benchmarking for the hydration of HCO_3_^–^, which was also studied by HPMS (see the Supporting Information). Theory consistently predicts a significant drop in water binding energy for the second and third water molecule, which is consistent with the chemical intuition, in stark contrast to the reported HPMS values summarized in Table S4. This suggests that the present experimental results based on BIRD and MEM under very well-controlled conditions are more reliable.

## Conclusions

In this work, the evaporation of water molecules due to blackbody infrared radiation has been investigated to determine the binding energies of CO_3_^·^^–^(H_2_O)_1,2_. BIRD kinetics were measured at different temperatures, yielding rate constants for different temperatures. The measurements show that a collision-free environment is essential for a quantitative evaluation by MEM. As a pressure limit, Dunbar suggested a pressure lower than 10^–6^ mbar for the observation of BIRD.^25^ For quantitative work, however, our results indicate that collisions at a pressure of 10^–9^ mbar can have an impact on the results, especially for small systems and long BIRD lifetimes. This shows that the UHV pressure should be as low as possible for reliable BIRD experiments, and that the pressure for which collisions have an impact on the dissociation rate depends strongly on the properties of the studied system.

The experimental BIRD kinetics were fitted with MEM. We introduce a multiple-well approach, which yields hydration energies at *T* = 0 K of CO_3_^·^^–^ of 56 ± 6 kJ/mol for the first and 45 ± 3 kJ/mol for the second water molecule. These results are in very good agreement with high-level ab initio calculations. This indicates that the used approach works well for the description of the temperature dependence of the evaporation of water molecules due to blackbody radiation.

In the MEM, an empirical scaling factor, *s,* is used for infrared absorption intensities. This factor does not change the slope of the Arrhenius plot but leads to an offset of the linear fit. The deviation of the scaling factor from 1 is in part due to the harmonic approximation used for the vibrational modes. Collisions, however, also increase the rate of energy exchange with the environment, and high scaling factors may hint at their presence. Already for CO_3_^·^^–^(H_2_O)_2_, which features four low-lying isomers, the multiple-well approach yields better results than the single-well approach since it better describes the actual situation in the experiment.

## Supporting Information Available

Arrhenius plot with all experimental data; resulting MEM activation energies for alternative approaches; reaction energies for water dissociation and interconversion between various isomers; calculated binding enthalpies for hydration of CO_3_^·–^ and HCO_3_^–^ compared with literature values; comparison of MEM results with different activation energies and optimized empirical scaling factors *s*; and Cartesian coordinates and electronic energies of calculated isomers (PDF)
Original data shown in Figures 2, 3, 5: raw mass spectrometry data Fig 2a and 2b (XLSX);
experimental rates and rates from master equation modeling: Fig 3a and 3b (XLSX); and
population of isomers and energy levels of both multiple-well and single well approaches: Fig 5a, 5b, 5c, and 5d (XLSX)
