# Supplementary material for: Simplified Multiple-Well Approach for the Master Equation Modeling of Blackbody Infrared Radiative Dissociation of Hydrated Carbonate Radical Anions
Source: J Am Chem Soc. 2022 Nov 16;144(47):21485–93. doi: 10.1021/jacs.2c07060 (PMC9716553; doi:10.1021/jacs.2c07060)
Supplement: Supplementary file 1 — ja2c07060_si_001.pdf [file ja2c07060_si_001.pdf]

Supporting Information for

A simplified multiple-well approach for the master equation modelling of blackbody infrared radiative dissociation (BIRD) of hydrated carbonate radical anions

Magdalena Salzburger, Milan Ončák, Christian van der Linde, and Martin K. Beyer**

Institut für Ionenphysik und Angewandte Physik, Universität Innsbruck, Technikerstraße 25, 6020 Innsbruck, Austria

E-mail: milan.oncak@uibk.ac.at; martin.beyer@uibk.ac.at

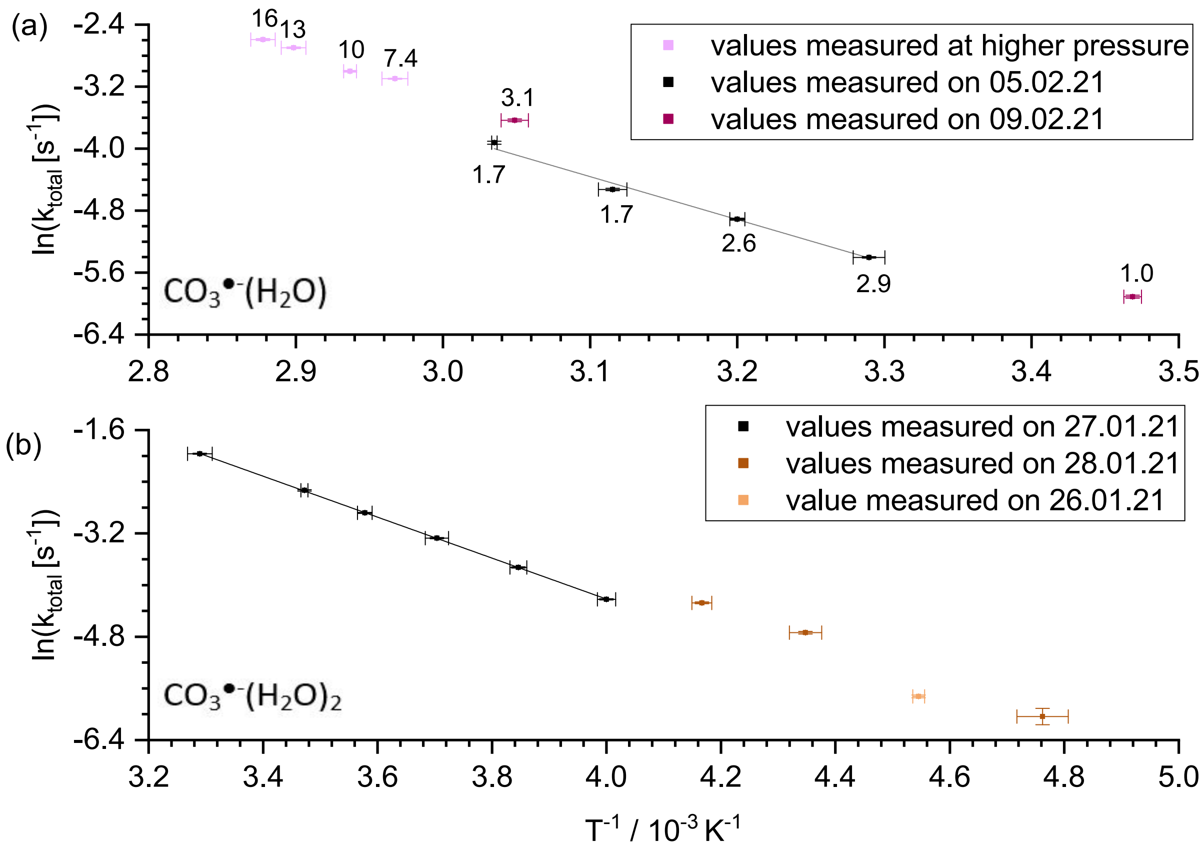

Figure S1: Arrhenius plot of experimental data: Black datapoints were used for further evaluation. Numbers next to the data points indicate the pressure in 10^{-10} mbar in the ICR cell during the respective measurement.

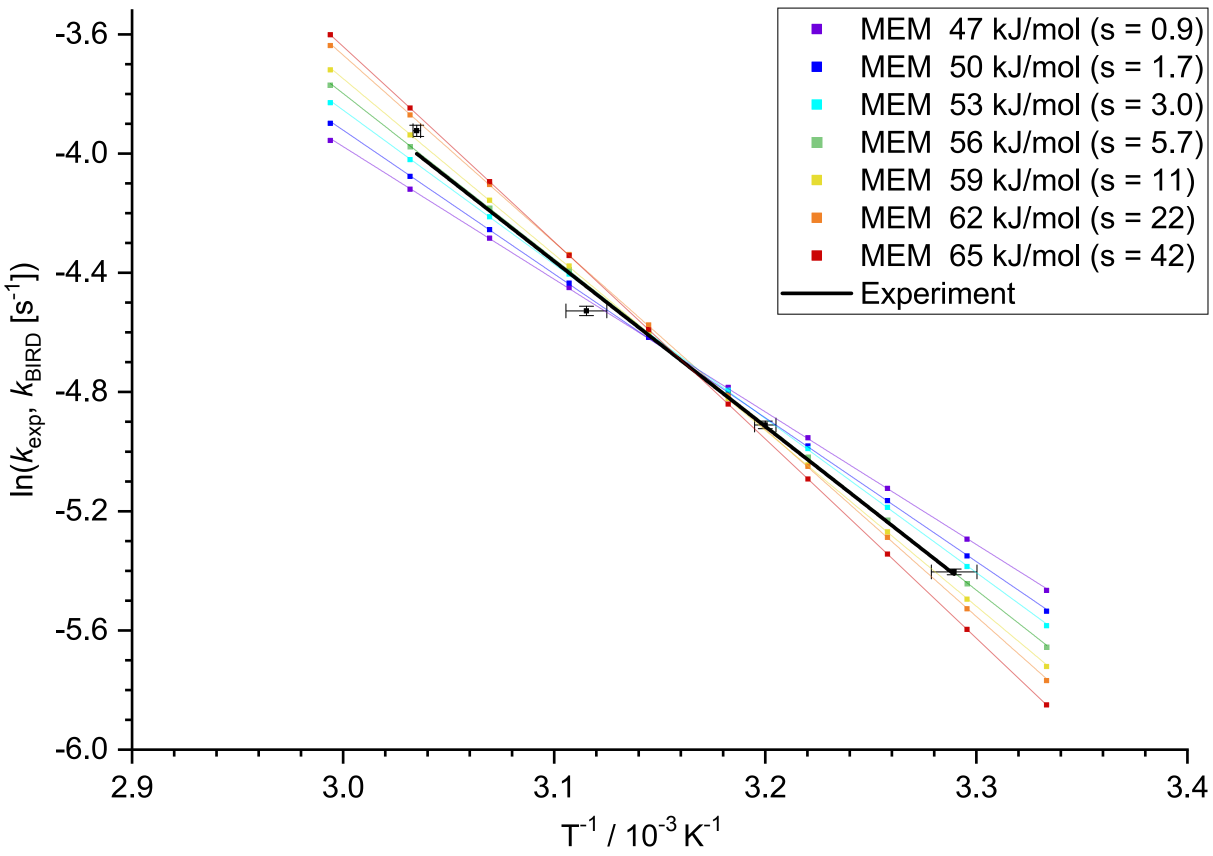

Figure S2: Comparison of MEM results with different activation energies and respectively adapted empirical scaling factors *s*.

Table S1: Resulting MEM activation energies $E^{\#}$ (in kJ/mol) if all data points (see Figure S1) are considered. The empirical scaling factor *s* is given in parentheses.

	$E^{\#}$	
	Single-well approach	Multiple-well approach
$CO_3^{\bullet -}(H_2O)$	65 (s = 5.4)	65 (s = 5.4)
$CO_3^{\bullet -}(H_2O)_2$	37 (s = 0.6)	39 (s = 0.6)

Table S2: Reaction energies (in kJ/mol) for water dissociation and interconversion between various isomers. See Figure 5 for isomer structures. Calculations were performed in structures optimized at the CCSD/aug-cc-pVDZ level using the respective zero-point correction. "*X*Z" stands for aug-cc-pV*X*Z basis set. The complete basis set (CBS) value is gained through extrapolation in TZ, QZ series.

	CCSD/DZ	CCSD(T)/DZ	CCSD(T)/TZ	CCSD(T)/QZ	CCSD(T)/CBS
ia → CO_3^- + H_2O	55.2	55.2	55.5	55.0	53.6
ib → CO_3^- + H_2O	51.5	55.2	56.2	55.7	54.1
ia → ib	3.7	0.0	-0.7	-0.7	-0.5
iia → $CO_3^-H_2O$ + H_2O	46.2	47.7	47.1	45.9	42.2
iib → $CO_3^-H_2O$ + H_2O	45.3	47.5	47.0	46.3	44.2
iic → $CO_3^-H_2O$ + H_2O	41.2	43.5	43.4	42.7	40.5
iid → $CO_3^-H_2O$ + H_2O	39.8	41.4	40.4	39.8	38.0
iia → iib	0.9	0.2	0.0	-0.5	-2.0
iia → iic	5.0	4.2	3.6	3.1	1.7
iia → iid	6.3	6.3	6.7	6.0	4.2

Table S3: Resulting MEM activation $E^{\#}$ using relative energies calculated at the CCSD(T)/CBS //CCSD/aug-cc-pVDZ level (in kJ/mol). The empirical scaling factor *s* is given in parentheses.

	$E^{\#}$	
	Single-well approach	Multiple-well approach
$CO_3^{\bullet -}(H_2O)$	56 (s = 5.7)	56 (s = 5.8)
$CO_3^{\bullet -}(H_2O)_2$	42 (s = 1.2)	45 (s = 1.2)

Table S4: Binding energies ΔE (in kJ/mol) and enthalpies ΔH (in kJ/mol; at T = 298.15 K, p = 1 atm) for the first, second and third hydration of CO_3^- and HCO_3^- calculated at the B3LYP/aug-cc-pVDZ level compared with enthalpies measured by high-pressure mass spectrometry (HPMS) reported by Keesee *et al.* (*J. Am. Chem. Soc.* **1979**, *101*, 2599–2604). No frequency scaling was applied.

	ΔE	ΔH	HPMS
$CO_3^-H_2O \rightarrow CO_3^- + H_2O$	47	49	59
$CO_3^-(H_2O)_2 \rightarrow CO_3^-H_2O + H_2O$	42	45	57
$CO_3^-(H_2O)_3 \rightarrow CO_3^-(H_2O)_2 + H_2O$	39	43	55
$HCO_3^-H_2O \rightarrow HCO_3^- + H_2O$	61	64	66
$HCO_3^-(H_2O)_2 \rightarrow HCO_3^-H_2O + H_2O$	50	53	62
$HCO_3^-(H_2O)_3 \rightarrow HCO_3^-(H_2O)_2 + H_2O$	42	46	57

Cartesian coordinates (in Å) and electronic energies including the zero-point correction (in Hartree) of isomers optimized at the CCSD/aug-cc-pVDZ level

H2O
E = -76.247196
O -0.000000 -0.000000 0.118548
H 0.000000 -0.760651 -0.474193
H 0.000000 0.760651 -0.474193

CO3-
E = -263.206004
o 0.000000 0.000000 1.265619
c 0.000000 0.000000 -0.072973
o 0.000000 1.141629 -0.605445
o -0.000000 -1.141629 -0.605445

CO3-.H2O, ia
E = -339.474077
C 0.000000 0.000000 -0.716053
O 0.000000 -0.000000 -2.059271
O 0.000000 1.143613 -0.187243
O -0.000000 -1.143613 -0.187243
O 0.000000 -0.000000 2.505100
H 0.000000 0.730365 1.862789
H -0.000000 -0.730365 1.862789

CO3-.H2O, ib
E = -339.472695
C -0.000000 0.820197 0.000000
O 1.221921 0.315848 0.000000
O 0.075917 2.092164 0.000000
O -1.023554 0.094981 -0.000000
O -0.278120 -2.604100 -0.000000
H -0.638797 -1.688764 -0.000000
H 0.669486 -2.423562 0.000000

CO3-.(H2O)2, iia
E = -415.738684
C 1.233960 -0.004943 0.016031
O 0.739142 1.107194 0.333428
O 2.572753 0.010673 -0.080405
O 0.681075 -1.112730 -0.217736
H -2.281444 0.592978 -0.266454
O -2.078695 1.538837 -0.203176
H -1.119051 1.515910 -0.029494
H -2.095903 -1.378777 1.030795
O -2.021088 -1.464041 0.073468
H -1.052860 -1.339914 -0.075665

CO3-.(H2O)2, iib
E = -415.738413
C -0.033952 -0.695963 0.000000
O 0.909041 -1.531099 0.000000
O -0.000000 0.564612 -0.000000
O -1.234862 -1.287568 0.000000
O -2.580745 1.734594 -0.000000
O 2.929500 0.661120 -0.000000
H 2.718587 -0.285334 0.000000
H 2.020783 1.007716 -0.000000
H -3.066427 0.901616 -0.000000
H -1.652707 1.418517 -0.000000

CO3-.(H2O)2, iic
E = -415.736864
C 0.000000 0.000000 -0.541308
O -0.000000 -0.000000 0.800794
O 0.000000 1.140501 -1.071627
O -0.000000 -1.140501 -1.071627
O -0.000000 -3.364359 0.684367
O 0.000000 3.364359 0.684367
H -0.000000 2.857618 1.504548
H -0.000000 2.648739 0.014279
H -0.000000 -2.648739 0.014279
H -0.000000 -2.857618 1.504548

CO3-.(H2O)2, iid
E = -415.736286
C -0.345621 1.466220 0.000000
O -1.090453 2.584005 0.000000
O -0.052284 1.024933 1.143527
O -0.052284 1.024933 -1.143527
O 1.318054 -1.185754 -0.000000
O -0.052284 -3.628505 -0.000000
H 0.998934 -0.625639 -0.732285
H 0.998934 -0.625639 0.732285
H 0.469825 -2.797512 -0.000000
H -0.959965 -3.305419 -0.000000

Cartesian coordinates (in Å) and electronic energies including the zero-point correction (in Hartree) of isomers optimized at the B3LYP/aug-cc-pVDZ level

H2O
E = -76.423408
O 0.000000 0.000000 0.117845
H 0.000000 -0.763880 -0.471380
H 0.000000 0.763880 -0.471380

CO3-
E = -263.847134
o 0.000000 0.000000 1.277242
c 0.000000 -0.000000 -0.000067
o 0.000000 1.106166 -0.638596
o -0.000000 -1.106166 -0.638596

CO3-.H2O
E = -340.288412
C 0.000000 0.817386 -0.000000
O 1.222060 0.430014 -0.000000
O -0.160726 2.093888 -0.000000
O -0.980607 0.019553 0.000000
O -0.117198 -2.638221 0.000000
H -0.527601 -1.740861 0.000000
H 0.819374 -2.405327 0.000000

CO3-.(H2O)2
E = -416.727695
C 1.299293 -0.009150 0.011688
O 0.728304 1.105430 0.260338
O 2.583460 0.054284 -0.084730
O 0.694465 -1.110379 -0.131622
H -2.303182 0.541489 -0.189659
O -2.158047 1.503393 -0.147555
H -1.191539 1.550007 -0.029057
H -2.107095 -1.536500 0.965961
O -1.995537 -1.449894 0.012456
H -1.015103 -1.322767 -0.088466

CO3-(H2O)3
E = -493.165957
c 1.579698 -0.036833 -0.012552
o 0.741058 -0.997631 0.015599
o 2.831791 -0.330740 0.043640
o 1.259233 1.186142 -0.091798
o -1.519657 1.746290 -0.194395
o -1.789685 -1.018773 -1.354497
o -1.723330 -0.564012 1.594683
h -1.775481 0.344342 1.239444
h -0.825560 -0.829778 1.321655
h -1.733817 1.042298 -0.831261
h -0.541379 1.686855 -0.142053
h -2.163731 -1.123206 -0.463305
h -0.833505 -1.069715 -1.155030

HCO3-
E = -264.481600
C 0.000000 0.170626 -0.000000
O 1.230397 0.427619 -0.000000
O -1.005917 0.897583 -0.000000
O -0.297472 -1.247695 0.000000
H 0.583932 -1.643805 0.000000

HCO3-.H2O,
E = -340.928359
C -0.000000 0.620704 0.000000
O 1.154084 0.153017 0.000000
O -0.063526 2.043740 0.000000
O -1.123652 0.048274 0.000000
O 0.133805 -2.520039 0.000000
H -0.626545 -1.905997 0.000000
H 0.834628 -1.838607 0.000000
H -1.013770 2.220445 0.000000

HCO3-.(H2O)2,
E = -417.370940
C 1.149172 -0.031264 -0.004187
O 0.560111 -1.129627 -0.118370
O 0.701166 1.135517 0.111072
O 2.558423 -0.133848 -0.003518
O -2.097438 -1.427541 -0.011644
O -2.092467 1.564399 -0.071010
H -2.333979 0.626527 -0.156106
H -1.113599 1.505419 -0.003315
H -2.228462 -1.504511 0.939911
H -1.108647 -1.294130 -0.094037
H 2.851296 0.783077 0.086429

HCO3-(H2O)3,
E = -493.810528
C 1.435936 0.044821 -0.026608
O 2.808191 -0.237184 0.049173
O 1.147039 1.248054 -0.140038
O 0.680953 -0.974338 0.033060
O -1.568731 1.721175 -0.259763
O -1.707366 -0.495496 1.647753
H -1.808167 0.387513 1.242217
H -0.836124 -0.763939 1.294792
H -1.799574 1.005725 -0.874894
H -0.583116 1.640792 -0.209833
H 2.845305 -1.199674 0.133025
H -2.233092 -1.210718 -0.467367
O -1.779849 -1.140527 -1.321914
H -0.842739 -1.102096 -1.024464
